# Microfluidic Technology for the Production of Hybrid Nanomedicines

**DOI:** 10.3390/pharmaceutics13091495

**Published:** 2021-09-17

**Authors:** Ilaria Ottonelli, Jason Thomas Duskey, Arianna Rinaldi, Maria Vittoria Grazioli, Irene Parmeggiani, Maria Angela Vandelli, Leon Z. Wang, Robert K. Prud’homme, Giovanni Tosi, Barbara Ruozi

**Affiliations:** 1 Nanotech Lab, Te. Far.T.I., Department Life Sciences, University of Modena and Reggio Emilia, 41125 Modena, Italy; ilaria.ottonelli@unimore.it (I.O.); jasonthomas.duskey@unimore.it (J.T.D.); arianna.rinaldi@unimore.it (A.R.); vittoriagrazioli@unimore.it (M.V.G.); irene.parmeggiani@unimore.it (I.P.); mariaangela.vandelli@unimore.it (M.A.V.); barbara.ruozi@unimore.it (B.R.); 2 Clinical and Experimental Medicine Ph.D. Program, University of Modena and Reggio Emilia, 41125 Modena, Italy; 3 Department of Chemical and Biological Engineering, Princeton University, Princeton, NJ 08544, USA; lzwang@princeton.edu (L.Z.W.); prudhomm@princeton.edu (R.K.P.)

**Keywords:** nanomedicine, hybrid nanoparticles, nanoprecipitation, microfluidics

## Abstract

Microfluidic technologies have recently been applied as innovative methods for the production of a variety of nanomedicines (NMeds), demonstrating their potential on a global scale. The capacity to precisely control variables, such as the flow rate ratio, temperature, total flow rate, etc., allows for greater tunability of the NMed systems that are more standardized and automated than the ones obtained by well-known benchtop protocols. However, it is a crucial aspect to be able to obtain NMeds with the same characteristics of the previously optimized ones. In this study, we focused on the transfer of a production protocol for hybrid NMeds (H-NMeds) consisting of PLGA, Cholesterol, and Pluronic^®^ F68 from a benchtop nanoprecipitation method to a microfluidic device. For this aim, we modified parameters such as the flow rate ratio, the concentration of core materials in the organic phase, and the ratio between PLGA and Cholesterol in the feeding organic phase. Outputs analysed were the chemico–physical properties, such as size, PDI, and surface charge, the composition in terms of %Cholesterol and residual %Pluronic^®^ F68, their stability to lyophilization, and the morphology via atomic force and electron microscopy. On the basis of the results, even if microfluidic technology is one of the unique procedures to obtain industrial production of NMeds, we demonstrated that the translation from a benchtop method to a microfluidic one is not a simple transfer of already established parameters, with several variables to be taken into account and to be optimized.

## 1. Introduction

Nanomedicine has been the leading topic of interest for novel therapeutic approaches against difficult-to-treat diseases for the last decade [1] thanks to the possibility of loading drugs that are normally inaccessible for direct injection due to their poor solubility, increasing the pharmacokinetic half-life of drugs in the blood and even decreasing off-target effects or of exploiting targeting ligands to engineer NMed surfaces for selective and tailored treatments. In particular, the potential of NMeds has been recently highlighted by the development of the nanomedicine-based vaccine against COVID-19 [2,3,4]. This incredible scientific effort has pointed out more than before that the ability to control the design and production of nanomedicines (NMed) is a crucial aspect for their eventual success with strong chances of ameliorating therapeutic effects.

In fact, notwithstanding all the promising results in the field, few NMeds have passed the rigorous selection required for commercial availability [5]. A major reason for this bottleneck in commercially available NMeds lies in the difficulty of making classic benchtop methods reproducible or automated in a way that can be directly translated to large-scale industrial use. This passage is vital to ensure that industrial nanoproduction will allow the obtainment of NMed with standardized features, thus allowing governing agencies, such as the Food and Drug Administration (FDA) or European Medicines Agency (EMA), to certify them for commercial human use. In fact, the variations in fundamental parameters of optimized small-scale benchtop methods to those that allow reproducibility at a large scale must not affect the critical NMed pharmaceutical characteristics that may define their success, such as: a size ranging from 100–400 nm for improved biodistribution, surface charge minimizing first past clearance as well as the potential aggregation process, and standard drug content [6,7,8].

To combat this issue, companies have increased the investigations and optimizations of microfluidic systems [9,10]. These devices allow the reproducible production of NMeds with increased consistency thanks to the automated and constant output of a standardized and certified machine [11,12] resulting in NMeds with consistent physical characteristics and drug loading. Moreso, they open up a direct path for upscaling through higher yield, minimized production time, and much larger production volumes with minimal or no batch variability. The result of the application of microfluidics is a certifiable and FDA-approved vaccine rolled out worldwide, proving the potential of a successful NMed design that passed phase 4 clinical trials and entered production on a global scale [13,14].

Even with its several advantages, the use of microfluidic devices for the production of NMeds requires an in-depth optimization of the protocols and instrumentation settings. For any research laboratory, but most of all for small university laboratories, far from the economic possibilities of big companies such as Pfizer or Moderna, the application of microfluidics opens two different scenarios: (1) the use of microfluidic devices for the design and optimization of NMeds directly from a small scale or (2) the adaptation of already established small-scale benchtop protocols to a microfluidic system. In the first case, the use of microfluidic devices from the beginning during the small-scale NMed design ensures that the protocols are compatible and always reproducible as the same technology is used throughout the process. At the same time, this approach has a high upfront cost linked to the cost of the machine, and the proprietary and often mono-use cartridges used for each individual sample; even non-commercialized, 3D-printed or home-made devices can have a difficult set-up and several requirements. More importantly, this first approach is only possible when considering the design of novel NMeds; it cannot be applied to the numerous well-established or already published successful NMeds. In an evolving scenario where microfluidic devices for NMed production are taking the spotlight as a new paradigm instead of classical nanoprecipitation [15], it would be wasteful to abandon all the previous optimization studies to restart completely with a microfluidic process and incur all the upfront costs. This second one is a more classical and maybe economical approach that is used to translate optimized small-scale production of NMeds to a microfluidic system; however, adapting benchtop protocols to a microfluidic device to obtain NMeds similar to the well-known ones can be a difficult and time-consuming process [16,17].

Microfluidic systems have already been tested for the translation and automated production of already established polymeric [18,19,20,21] and lipidic [22,23,24,25,26,27] NMed systems, as well as some hybrid [28,29,30,31] nanomedicines consisting of a polymeric core and a lipidic shell, which are now abundant in the literature. However, some new and innovative hybrid NMeds are yet to be optimized to exploit microfluidics for their improved production. Therefore, in this work we studied the transfer to a microfluidic-based protocol of a well-optimized and recently published [32,33] formulation method for hybrid nanomedicines (H-NMeds), consisting of the FDA-approved polymer poly-lactide-co-glycolic acid (PLGA) and cholesterol (Chol), a biocompatible and ubiquitous molecule [34] widely used for NMed formulation [35,36,37,38]. To this end, attempts to translate the benchtop production of H-NMed to this automated and standardized technology were performed by exploiting a homemade 2–channel microfluidic device.

The aim was to assess whether this already optimized benchtop method was easily transferable to the microfluidic technology by comparing physical and compositional changes, as well as morphological features, between the well-known H-NMeds from benchtop protocols and those reproduced with the microfluidic device. The parameters varied to attempt and obtain comparable H-NMeds were (1) initial concentration of core materials in the organic phase; (2) the flow rate ratio (FRR, the ratio of the aqueous to organic phase); (3) the ratio of polymer to cholesterol. The resulting H-NMeds were then compared in terms of size and homogeneity, surface charge, morphology, composition, and storage stability.

## 2. Materials and Methods

### 2.1. Materials

Poly (d,l-lactide-co-glycolide) (PLGA, RG503H,MW ≅ 11,000) was used as received from the manufacturer (Evonik, Essen, Germany). Isopropanol was purchased from Carlo Erba, Cornaredo Milan, Italy. Cholesterol, Pluronic^®^ F68, Acetonitrile, Ethanol, Acetone, Chloroform, Barium Chloride (BaCl_2_), Iodide (I_2_), and Potassium Iodide (KI) were purchased from Sigma Aldrich (Merck Life Sciences, Milan, Italy). All solvents and reagents purchased were of analytical purity and used as delivered.

### 2.2. H-NMed Formation by the Optimized Benchtop Method

H-NMeds were obtained by adapting an already optimized benchtop protocol [32,33] with minor modifications: 20 mg of Chol and 20 mg of PLGA were weighed and dissolved in 4 mL of acetone. This organic phase was then added dropwise into a beaker containing 50 mL of a 5 mg/mL Pluronic^®^ F68 solution warmed at 45 °C and kept under magnetic stirring. After 15 min, the suspension was left for 1 h at room temperature and then the solvent was removed under vacuum via Rotavapor^®^ for 30 min. The obtained H-NMeds were purified by centrifugation at 14,500 rpm for 10 min at 4 °C. The supernatant was discarded, and the pellet was resuspended in 4 mL of MilliQ (Millipore, Bedford, MA, USA). From the resulting suspension, 10 µL was diluted in 1 mL of MilliQ water for size, zeta potential, and microscopy studies. Another 500 µL aliquot of H-NMed suspension was lyophilized for weight yield and compositional analysis.

### 2.3. Optimization of H-NMed Formation by Microfluidics

To optimize the microfluidic method, a similar protocol to the benchtop one was used: PLGA and Cholesterol were dissolved in acetone and mixed with a 5 mg/mL Pluronic^®^ F68 solution at 45 °C in the microfluidic device to produce H-NMeds, using a Total Flow Ratio of 10 mL/min. The temperature, composition of the aqueous phase, and Total Flow Ratio were maintained constant throughout the experiments, as well as the organic solvent, in order to ensure high diffusibility with the organic phase and therefore a fine mixing of the two in the device. The parameters changed to reach an optimization of this method included: (1) the ratio (*v*:*v*) of organic and aqueous solutions (from 12, 5:1 to 1:1) with final volume kept constant at 13 mL; (2) total concentration of PLGA and Cholesterol in the organic solution (from 5 to 30 mg/mL); (3) ratio of PLGA to Cholesterol in the organic phase (*w*:*w*, from totally polymeric 100:0 to totally lipidic 0:100), keeping a total concentration of materials of 20 mg/mL. After formulation through the microfluidic device, NMeds were left under magnetic stirring at room temperature for 2 h to allow solvent evaporation. The final suspension was centrifuged at 14,500 rpm for 10 min and resuspended in MilliQ, and aliquots of 2 mL of suspension were lyophilized for weight yield and compositional analysis.

### 2.4. Size and Surface Charge Analysis

The mean particle size (Z-Average) and polydispersity index (PDI) of all samples were determined by Photon Correlation Spectroscopy (PCS) analysis, using a Zetasizer Nano ZS (Malvern Panalytical, Malvern, UK; Laser 4 mW He-Ne, 633 nm, Laser attenuator Automatic, transmission 100–0.0003%, Detector Avalanche photodiode, Q.E. > 50% at 633 nm, T = 25 °C). All samples were diluted before being analyzed to arrive at a final concentration of ~0.1 mg/mL. All data are expressed as the means of at least three individual H-NMed preparations.

The zeta potential (ζ-pot) was measured using a Zetasizer Nano ZS (Malvern Panalytical, Malvern, UK) with a combination of laser Doppler velocimetry and a patented phase analysis light scattering method (M3-PALS). The same samples subjected to PCS (0.1 mg/ mL) were analyzed using DTS1070 zeta potential cuvettes and expressed as the mean of at least three individual H-NMed preparations.

### 2.5. Weight Yield

Aliquots of a water suspension of purified H-NMeds were freeze-dried (−60 °C, 1 × 10^−3^ mm/Hg; LyoLab 3000, x-Holten, Allerod, Denmark) for at least 8 h and weighed. The yield (WY %) was calculated as follows:

WY(%) = ((mg of freeze-dried sample)/(mg PLGA + mg cholesterol)) × 100(1)

### 2.6. Quantification of Cholesterol

An aliquot of lyophilized H-NMeds (~1 mg) was dissolved in 300 µL of chloroform. After sonication and vortex for 60 s, 600 µL of isopropanol was added and the solution was vortexed again. The solvent mixture was put under magnetic stirring to evaporate the chloroform and precipitate the PLGA in the isopropanol phase. Isopropanol was eventually adjusted to a final volume of 1 mL. This solution was centrifuged at 13,000 rpm for 10 min. The supernatant was analysed by HPLC using a Syncronis C18 4.6 × 250 mm 5 µm reverse phase column using an isocratic gradient of 50:50 ethanol (EtOH) absolute and acetonitrile (ACN) and a flow rate of 1.2 mL/min. The retention time of cholesterol was 16 min and had a linear range from 50 to 1000 µg/mL at 210 nm (Curve: y = 2545.70x − 32555.1: R^2^ = 0.994596). The total cholesterol content was calculated based on three injections from different formulations.

The amount of cholesterol in the formulations was calculated as Chol Recovery % (CR %) and Cholesterol Content % (CC %) with the following formulas:

CR(%) = ((mg of Chol in the formulation)/(mg of Chol used for formulation)) × 100(2)

CC(%) = ((mg of Chol in the formulation)/(mg of H-NMeds analysed)) × 100(3)

### 2.7. Quantification of Residual Pluronic^®^ F68

The residual amount of surfactant in the H-NMeds was determined by a colorimetric method [39]. Briefly, ~1 mg of a freeze-dried H-NMeds sample was solubilized in 0.5 mL of dichloromethane. Then, 10 mL of distilled water was added and the organic solvent was evaporated at r.t. under stirring for 2 h. The suspension was filtered (cellulose acetate filter, porosity 0.45 µm, Sartorius, Florence, Italy) to obtain an aqueous solution (A).

To calculate the amount of Pluronic^®^ F68 in the formulation, 2 mL of the aqueous solution (A) was treated with 2 mL of 0.5% (*w*/*v*) BaCl_2_ in HCl 1 N and 0.5 mL of an aqueous solution of I_2_/KI (0.05 M/0.15 M). The obtained solution was incubated at r.t. for exactly 10 min in the dark. Pluronic^®^ F68 concentration was determined measuring the absorbance at 540 nm (Model V530, Jasco, Cremella, Italy). A calibration curve was calculated using the same method on stock solutions of Pluronic^®^ F68, and linearity was found in the range of 2–18 µg/mL. All data are expressed as the mean of at least three determinations. The amount of surfactant in the formulation was expressed as the Pluronic^®^ F68 Content % (PC %) and calculated using the following equation:

PC(%) = ((mg of Pluronic^®^ F68 in the formulation)/(mg of H-NMeds analysed)) × 100 (4)

### 2.8. Morphological Studies

AFM observations were performed with an atomic force microscope (Park Instruments, Sunnyvale, CA, USA) at about 25 °C operating in air and in non-contact mode using a commercial silicon tip-cantilever (high-resolution noncontact “GOLDEN” Silicon Cantilevers NSG–11, NT-MDT, tip diameter 5–10 nm; Zelenograd, Moscow, Russia) with stiffness of about 40 Nm^–1^ and a resonance frequency of around 150 kHz. After the purification, the sample was dispersed in distilled water (0.01 mg/mL) before being applied to a freshly cleaved mica disk (1 cm × 1 cm); two minutes after the deposition, the excess water was removed using a blotting paper. The AFM images were obtained with a scan rate 1 Hz. Two kinds of images were obtained: the first one was a topographical image and the second one was indicated as “error signal”. This error signal was obtained by comparing two signals: the first one, direct, representing the amplitude of the vibrations of the cantilever, and the other one being the amplitude of a reference point. The images obtained by this method showed small superficial variations of the samples. Images were processed using ProScan Data Acquisition software (Park Instruments, Sunnyvale, CA, USA).

The structure of the samples was also analyzed by scanning transmission electron microscopy (STEM FEI Nova NanoSEM 450, Bruker, Billerica, MA, USA). Briefly, a drop of the same water-diluted suspension (0.01 mg/mL) used for AFM imaging was placed on a 200–mesh copper grid (TABB Laboratories Equipment, Berks, UK), allowed to adsorb, and the suspension surplus was removed by filter paper. All grids were analyzed using the transmission electron microscope operating at 25 kV using a STEM II detector in Field free mode.

### 2.9. Storage Stability

Aliquots of 10 μL of H-NMed formulation at a final concentration of 10 mg/mL were tested for stability to lyophilization and freezing. In particular, the microfluidic formulation that was found to be the most similar in composition and physical characteristics to the benchtop one was selected and used.

Samples were tested after addition to the suspension of different amounts of trehalose as a cryoprotectant, in *w*:*w* ratio with H-NMeds of 0:1 (no trehalose), 1:1, 3:1 and 6:1. Samples were vortexed for 60 s to allow solubilization of the sugar. Two freezing methods were used, namely, standard slow freezing at –19 °C and flash freezing by immersion of the aliquots in a dry ice and methanol bath until completely frozen. Once frozen, all these samples were lyophilized for 8 h and stored at + 4 °C until further analysis.

Another set of samples was prepared exactly as described above. Instead of lyophilization, samples were stored for one week at –19 °C independently from the freezing method used and then thawed at room temperature before further analysis.

Lyophilized or thawed samples were resuspended or diluted with 1 mL MilliQ, vortexed for 60 s, and eventually analyzed via PCS for size distribution and PDI, as previously described (Section 2.4).

### 2.10. Statistical Analysis

Statistical analysis was performed using Student’s T Test, where * *p* < 0.05 and ** *p* < 0.01, using the software GraphPad Prism 6 (GraphPad Holdings, San Diego, CA, USA). All samples were performed with *n* > 3, and the error bars in graphs indicate the standard deviation (SD) from the average.

## 3. Results and Discussion

Microfluidic systems have been studied and optimized over the last 20 years for their application for the production of nanomedicines (NMeds), showing advantages over traditional benchtop methods, such as higher reproducibility, batch to batch standardization, and direct translation towards industrial scale-up, as strongly emphasised by the recent production of the COVID-19 vaccine. Notwithstanding the undoubted benefits of the increased use of microfluidic based systems for the optimization of novel NMeds, the passage from traditional methods to this novel technology requires the careful adaptation of already optimized and published successful platforms without losing the features of the original NMeds. In this study, we propose the first optimization steps to adapt the production protocol of a well-established hybrid NMed consisting of PLGA and Cholesterol (H-NMed) and analysing how changing crucial parameters of a microfluidic-based process affects the physical and compositional characteristics of the resulting H-NMeds.

**Figure 1:**
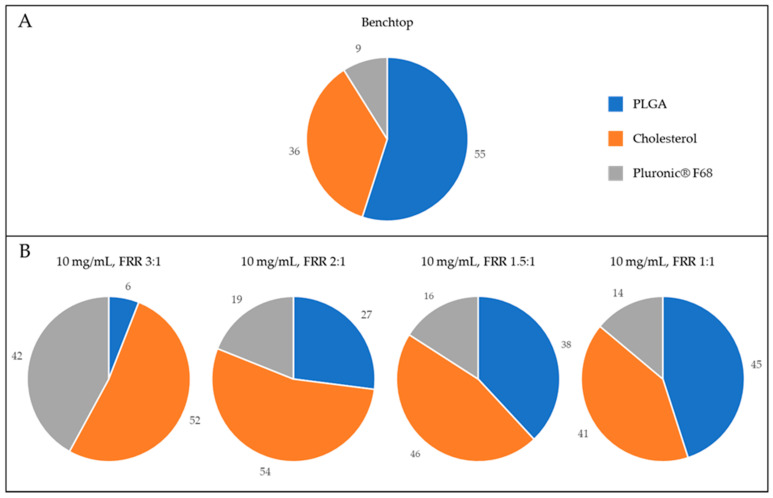
Graphical representation of the composition of H-NMeds. Blue: PLGA content. Orange: Cholesterol content. Grey: Pluronic^®^ F68 content. (**A**) H-NMeds obtained with nanoprecipitation. (**B**) H-NMeds obtained with microfluidic-based protocols using a concentration of materials in the organic phase of 10 mg/mL and different FRRs, namely, 3:1 (**left**), 2:1 (**center**) and 1:1 (**right**).

As a point of reference, H-NMeds produced with the classical benchtop protocol were analysed for size, homogeneity, surface charge, and composition (Table 1). Hybrid NMeds produced by nanoprecipitation are known [32] to have a final composition of about 30% Chol and to incorporate within their matrix about 10% of surfactant from the formulation environment (Figure 1A). They display a homogeneous size around 250 nm, with a strong negative surface charge around –35 mV. The published nanoprecipitation protocol used for these H-NMeds was extensively optimized [32,40], allowing for a high yield of almost 80%.

To start the optimization process using microfluidic technology, the concentration of materials in the organic phase, 10 mg/mL corresponding to that for nanoprecipitation, and the total volume (13 mL) were held constant. Another point to be considered prior to optimization is the surfactant since the microfluidic technology for NMed preparations is based on the fine mixing of two different fluids, namely, an organic and an aqueous solution, and the type and concentration of surfactant are crucial parameters to be addressed. This is valid not only for droplet microfluidics, now widely applied to high-throughput screenings [41,42,43,44,45], but also for NMed preparations. Triblock polymers such as Pluronics^®^ have been demonstrated to be safe and biocompatible [46,47] and are frequently used as stabilizers during the formation of NMeds both in benchtop [48,49,50,51] and in microfluidic based protocols [52,53,54,55]. For this reason, we standardized the use of Pluronic^®^ F68 as already exploited in our optimized benchtop methods to be used with the microfluidic device. Literature showed that a Pluronic^®^ F68 concentration between 0.1 and 1% leads to the successful formation of NMeds with microfluidic devices [56,57]; therefore, we fixed its concentration at 0.5% *w*/*v*, the same used in previous nanoprecipitation protocols.

### 3.1. Variation of the Flow Rate Ratio

With these variables fixed, the impact of changing the ratio between the volume of the aqueous and organic phases (Flow Rate Ratio, FRR) from 12.5:1 to 1:1 was analysed (Table 2). Analysis of the physico-chemical characteristics of these formulations showed an inverse trend in the average size of these H-NMeds, which increased from 170 to 250 nm when decreasing the FRR from 6:1 to 1:1 following literature reports for polymeric, lipidic, and other hybrid nanoparticles [16,18,58,59]. The only exception to this trend was represented by the formulation produced with the highest FRR of 12.5:1, where NMeds produced showed a higher poly-dispersity index (PDI > 0.3) and size over 250 nm. A deeper analysis of this formulation using Photon Correlation Spectroscopy prior to purification revealed the presence of a second population of particles with an average size of 70 nm accounting for almost 10% of the total intensity. This abundant subpopulation was hypothesised to be surfactant micelles [60] that could interfere with the proper formation of the PLGA and Chol into an H-NMed, leading to the low weight yield of 24% after purification, due to the poor pelleting of the small surfactant micelles.

On the other hand, the size distribution, PDI, and surface charge of every other formulation demonstrated the possibility to successfully formulate H-NMeds with various FRRs with similar physical characteristics to those created with the benchtop method; however, evident differences were found in their composition. In particular, analysing the amount of surfactant stably connected to the H-NMeds, a decreasing trend could be observed with the decreased FRR ranging from over 70% to a minimum of about 15%. Specifically, the selection of an FRR of 6:1 and 3:1 resulted in structures with a very high amount of surfactant, hinting towards the formation of particles with a different architecture with respect to the H-NMeds obtained by nanoprecipitation. Formulations produced with FRRs lower than 2:1 were the most similar to the classic benchtop H-NMeds, with a Pluronic^®^ F68 content of less than 20%, and 40–50% of the matrix being composed of Cholesterol (Figure 1B).

Nevertheless, the formulation with FRR 2:1 was the only one that showed the formation of homogeneous and monodispersed H-NMeds, as decreasing the FRR to 1.5:1 or 1:1 led to samples with a very high PDI > 0.4, which is generally considered a cutoff to determine whether a sample is homogeneous in size [61], and a higher variability in size. Additionally, the formulation with FRR or 2:1 also showed the highest recovery of Cholesterol, almost 80%, hinting towards a lower loss of materials, a crucial point for an industrially relevant environment. For these reasons, H-NMeds produced with an FRR of 2:1 were deemed to be the most promising to be further optimized.

### 3.2. Variation in the Total Concentration of the Starting Material in the Organic Phase

The second step of the investigation on nanoproduction optimization was performed using a constant FRR of 2:1 and varying the total concentration of materials in the organic phase. Previous studies have pointed out an interesting relationship between the concentration of polymers or lipids in the organic phase and the size of the resulting NMeds, where an increase in their concentration of starting materials produces bigger polymeric NMeds [16,56,61,62] but smaller liposomes [63]. Regarding other types of NMeds consisting of both polymer and lipids, it is difficult to find information in the literature data that could help predicting the behaviour of our H-NMeds, as those are often formulated with dissolving phospholipid derivatives in the aqueous phase, and separately modifying the concentration of the polymer or the lipid [12,28,64,65,66,67,68] eventually leads to the same trends reported for single components. As reported in Table 3, each formulation resulted in an average size between 250 and 300 nm, with PDI < 0.3 and strongly negative surface charges of almost −30 mV. Nonetheless, it was possible to observe an inverse trend where the size decreased with an increased concentration from 5 to 30 mg/mL. This behavior could be attributed to a stronger influence of the lipidic component, which was calculated to account for slightly more than 50% of the total composition of the H-NMeds. Looking at the composition of these formulations, the amount of Pluronic^®^ F68 stably connected to the H-NMeds followed a trend where the Pluronic^®^ F68 decreased from 35 to 10% when the concentration of the core materials in the organic phase was increased. As previously mentioned, the residual surfactant stably associated with the matrix of an NMed is a crucial parameter to be evaluated. As reported in the literature, the type and surfactant not only influence the formation of the NMeds depending on their characteristics, such as HLB, molecular structure, and critical micelle concentration, but can also determine colloid stability: in fact, the concentration of the surfactant in the medium has an optimum, over which colloid stability decreases. Lastly, it impacts the interaction of NMeds with biological environments, as it can induce the formation of a protein corona with a different composition [69,70,71,72,73]. Despite their important role, surfactants are often considered secondary components of NMeds and remain unquantified. Here, we report a significant reduction in the amount of residual surfactant in these H-NMeds (* *p* < 0.02) by only varying the concentration of other core materials, underlining the importance of their quantification when optimizing a formulation protocol.

**Figure 2:**
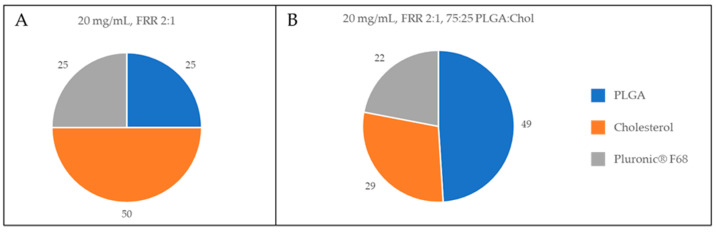
Graphical representation of the composition of H-NMeds. Blue: PLGA content. Orange: Cholesterol content (CC %). Grey: Pluronic^®^ F68 content. (**A**) H-NMeds obtained with the microfluidic device at a concentration of 20 mg/mL and an FRR of 2:1. (**B**) H-NMeds obtained with microfluidic-based protocols using a ratio of PLGA:Chol of 75:25, using an initial concentration of 20 mg/mL, and an FRR of 2:1.

Globally, no critical differences were found among these formulations in weight yield, Cholesterol content or physical characteristics. Considering the composition, the most similar H-NMed to our reference was the one obtained at the highest concentration of 30 mg/mL. Nevertheless, this concentration was experimentally found to be at the solubility limit of the PLGA and Cholesterol mixture, leading to a higher variability both in physical characteristics and composition as evidenced by the higher SD values for these results. Therefore, we decided to subject to further optimization the formulation produced with an initial concentration of 20 mg/mL (Figure 2A).

### 3.3. Variation of the PLGA: Cholesterol Ratio

The next step to optimize a microfluidic based protocol to produce H-NMeds was to test the possibility to use different ratios between PLGA and Cholesterol in the stock organic phase. To do this, H-NMeds were produced with a set FRR of 2:1 and a total concentration of materials of 20 mg/mL, ranging from a fully polymeric NMed of 100% PLGA to a fully lipidic one of 100% Cholesterol (Table 4).

PCS analysis revealed that it was possible to formulate NMeds at each ratio tested, with average size less than 400 nm, low PDI, and a strongly negative surface charge. However, it is clearly evident that a high presence of lipid in the organic phase correlates with an increase in the size, while an increase in the polymeric concentration corresponds to a size reduction. This evidence is in contrast to the trends reported in the literature and described above, in which polymers or lipids are used alone or in separate phases. In fact, these data suggest, without any literature precedent, a unique behavior of these H-NMeds, different from more simple situations where the interaction between polymeric chains and lipidic molecules dissolved in the same organic phase produces a novel effect.

At the same time, WY % also showed a significantly decreasing trend (* *p* < 0.05) related to an increase in the lipidic fraction used in the organic phase. This could be correlated with a different density of fully polymeric NMeds compared to fully lipidic ones, leading to a diverse reaction to the centrifugal forces applied during purification [74]. However, the lowest value of WY % reported was still not significantly different (*p* = 0.09) from that obtained with the original nanoprecipitation method (64 ± 6 vs. 77 ± 8%).

Compositional analysis revealed that the amount of Pluronic^®^ F68 that was stably recovered with the NMeds was independent from the variation of the organic phase composition and remained constant at around 20%, indicating that this value, from a process point of view, was more intimately linked to the FRR (Table 2) and total concentration of material in the organic phase (Table 3) during microfluidic formulations. Interestingly an increase in the Chol concentration in the stock organic phase did not correlate with a significant increase in its final content. In fact, when 75% of Cholesterol was used in the initial phase, the recovered NMeds showed a composition of around 50% of Cholesterol, despite the much higher initial concentration. This finding is again describing a difference in comparison to previous reports [40] in which the H-NMed obtained with benchtop nanoprecipitation procedures was shown to roughly keep within the NMed composition the cholesterol content from starting to the final step, confirming that changing the technology of production (from benchtop to microfluidic) impacted the composition of produced NMeds [75].

Among the formulations in Table 4, interestingly the PLGA:Chol ratio of 75:25 revealed the composition most similar to the composition of the benchtop-derived H-NMeds (with a similar Chol content % [29 vs. 36% (*p* = 0.17)], Cholesterol recovery Figure 1A and Figure 2B), and residual Pluronic^®^ F68 amounts (*p* = 0.09), although H-NMed obtained with microfluidics showed high variability in this last value (22 ± 10).

### 3.4. Stability Test and Morphology

**Figure 3:**
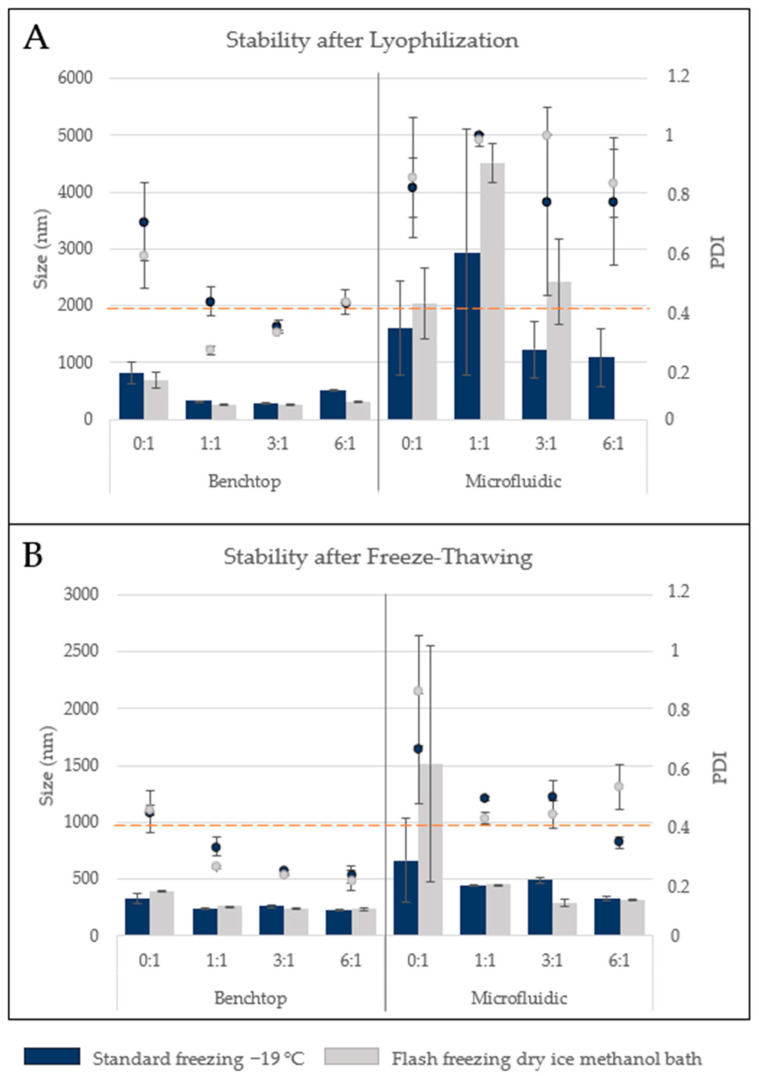
Size and homogeneity analysis of H-NMeds (**A**) after resuspension of lyophilized and (**B**) freeze-thawed aliquots. Bars: Size (nm), Dots: PDI. Blue bars and dots: standard freezing −19 °C, Grey bars and dots: flash freezing in a dry ice methanol bath. Each value is expressed as the Mean ± SD of three independent formulations.

To further assess if the most similar formulation showed other similar characteristics, the two optimized H-NMeds were tested for storage stability. Storage stability is crucial for industrial and clinical use: a good storage stability allows for easier industrial production and transport of therapeutics, while the lack of storage stability implies that the product has to be formulated the same day of administration, increasing human error and variability. H-NMeds from benchtop protocols are known to be stable to lyophilization; therefore, samples of benchtop-and microfluidic-derived H-NMeds were lyophilized and tested for size and homogeneity analysis after resuspension. Moreover, different amounts of cryoprotectant were added, from 0 to 6 times the weight of the H-NMeds in the aliquots tested. The method of freezing was also varied, using a standard slow freezing method and flash freezing by immersion in a dry ice and methanol bath (Figure 3A).

Data analysed revealed an evident difference between the H-NMeds from the two formulation techniques. Benchtop-formulated NMeds showed little to no aggregation after resuspension, especially with the addition of trehalose at a 1:1 or 3:1 ratio, with a size remaining under 300 nm and PDI lower than 0.4 independently from the freezing method. On the other hand, H-NMeds obtained with the microfluidic technology showed poor resuspension, with the formation of aggregates in the micrometric range and PDI close to 1 in all cases tested. Due to the poor prospect of lyophilized storage, another set of aliquots was then tested with the same variables for storing the samples frozen to verify which of the two steps was so detrimental for H-NMeds stability (Figure 3B). In fact, this test revealed that freezing alone also led to the aggregation of microfluidic-derived H-NMeds, showing an increase in size over 1 µm and PDI higher than 0.4. Here too, H-NMeds produced via nanoprecipitation revealed a different behavior, with a smaller average size and most importantly a smaller PDI around 0.2, indicating good homogeneity, compared with their microfluidic counterparts. Moreover, these samples revealed a decreasing trend in PDI correlated with the increase in cryoprotectant used [76], as expected from literature data. Globally, these tests suggested that despite the two H-NMeds having a similar composition in material percentages, they still displayed crucial differences, probably in their architecture, that determined their different response to the same condition or process, especially in a stressing step as freezing and lyophilization.

**Figure 4:**
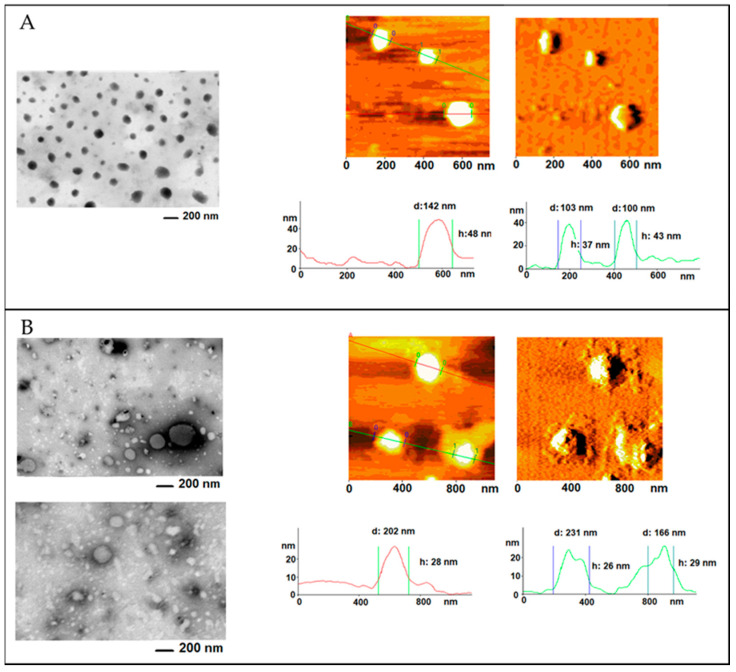
Microscopy images of H-NMeds produced via (**A**) nanoprecipitation or (**B**) the microfluidic device. (**Left** panels): STEM imaging. (**Right** panels): AFM imaging, topography, and error signal.

In order to furnish a view of the morphology of samples, both atomic force (AFM) and transmission microscopy (STEM) were conducted on the two different H-NMeds produced (Figure 4).

STEM analysis of benchtop-derived H-NMeds confirmed the presence of a homogeneous sample, as previously reported [40], where H-NMeds appeared spherical and monodisperse all across the sample. This was also confirmed by AFM images, where H-NMeds displayed a sharp border and a round shape, and it was possible to measure particle diameters of 100–150 nm. These images confirmed what was already demonstrated in previous studies [40], i.e., the formulation of a matrix of PLGA, Cholesterol, and Pluronic^®^ F68, where all components were strongly interconnected.

On the other hand, STEM images of microfluidic-derived H-NMeds revealed a sample with high variability, as evidenced by the two images reported in Figure 4B (left panel). This high variability was not recorded by PCS analysis, as this sample showed a low PDI of 0.26. Nevertheless, H-NMeds from of microfluidic production appeared to not be broadly uniform and therefore apparently less reproducible than the H-NMeds produced via nanoprecipitation. This variability in shape and size was also confirmed by AFM analysis: not only did the H-NMeds appear to be compressed and flattened under the tip of the cantilever, suggesting a softer structure, but the presence of unformed material surrounding the surface of formed H-NMeds was also evident. It was hypothesized that this material consisted of excess surfactant not stably connected to the surface of H-NMeds that was lost after deposition on the mica. Indeed, this is supported by the previous discussion regarding the quantification of Pluronic^®^ F68, which showed a higher presence of surfactant in this sample.

Taken together, these data suggest that the instability of the H-NMeds produced by microfluidics may be explained by an excess of Pluronic^®^ F68 that is not stably associated with or incorporated into the matrix of H-NMeds. This hypothesis is supported by past literature cited above, which confirmed that the amount of surfactant in the medium of NMeds plays a pivotal role in NMed stability, as it displays an optimum range of concentration, and an excess, especially if not strongly connected to the NMeds, could lead to instability due to its rearrangement into different structures [77,78].

## 4. Conclusions

In recent years, microfluidic technologies have taken the spotlight as a promising tool for the successful production of NMeds up to a global scale, as recently highlighted by the production of an NMed-based COVID19 vaccine. Nevertheless, the transition from established small-scale benchtop protocols to microfluidic devices faces several issues to produce NMeds with analogous features of those already optimized with benchtop protocols. In particular, several microfluidic parameters have to be taken into consideration, such as the flow rate ratio, concentration of core materials, and type of materials used, each of which could have an impact on NMed characteristics. Therefore, in this study, we aimed to investigate the translation of a well-known multi-component H-NMed consisting of PLGA and Cholesterol stabilized with Pluronic^®^ F68 from already established benchtop methods to a microfluidic device, in view of the possible exploitation of the unquestionable potential of microfluidic technology to standardize the production of these H-NMeds towards the high standards needed for GMP approval. Using an FRR of 2:1, a concentration of 20 mg/mL, and an initial ratio of PLGA:Cholesterol of 75:25, it was possible to reach the production of H-NMeds with statistically similar composition and chemico-physical properties to the benchtop ones, but still they displayed a critically different behavior when tested for storage stability. These data demonstrate that the translation of a multi-component system from an optimized benchtop method to a microfluidic-based system requires extensive efforts in terms of work and time in order to determine the optimal settings, not only during the microfluidic formulation, but also in the selection and amount of stabilizers, and methods for purification and storage, to ensure that the NMeds will have reproducible physico-chemical characteristics, composition, structures, and stabilities.

## Figures and Tables

**Table 1:** Physico-chemical characterization of H-NMeds obtained by the nanoprecipitation benchtop protocol. Standard Deviation (SD) is reported in parentheses.

[Materials]	FRR	Size (SD)	PDI (SD)	Zeta (SD)	Weight Yield % (SD)	Pluronic % (SD)	Chol Recovery % (SD)	Chol Content % (SD)
10 mg/mL	12.5:1	241 (14)	0.24 (0.03)	−36 (3)	77 (8)	9 (3)	50 (11)	36 (7)

**Table 2:** Physico-chemical characterization of H-NMeds obtained with the microfluidic device varying the Flow Rate Ratio (FRR). Standard Deviation (SD) is reported in parentheses.

[Materials]	FRR	Size (SD)	PDI (SD)	Zeta (SD)	Weight Yield % (SD)	Pluronic % (SD)	Chol Recovery % (SD)	Chol Content % (SD)
10 mg/mL	12.5:1	260 (45)	0.34 (0.04)	−27 (7)	24 (4)	/	/	/
10 mg/mL	6:1	173 (6)	0.27 (0.03)	−25 (5)	56 (6)	72 (13)	31 (2)	27 (1)
10 mg/mL	3:1	185 (0)	0.16 (0.02)	−26 (6)	49 (6)	42 (1)	51 (1)	52 (0)
10 mg/mL	2:1	262 (15)	0.19 (0.03)	−29 (5)	71 (3)	19 (3)	77 (6)	54 (4)
10 mg/mL	1.5:1	254 (52)	0.59 (0.04)	−28 (5)	53 (4)	16 (3)	41 (5)	46 (5)
10 mg/mL	1:1	245 (48)	0.57 (0.08)	−29 (6)	51 (2)	14 (1)	42 (2)	41 (2)

**Table 3:** Physico-chemical characterization of H-NMeds obtained with the microfluidic device varying the initial concentration of core materials in the organic phase. Standard Deviation (SD) is reported in parentheses.

[Materials]	FRR	Size (SD)	PDI (SD)	Zeta (SD)	Weight Yield % (SD)	Pluronic % (SD)	Chol Recovery % (SD)	Chol Content % (SD)
5 mg/mL	2:1	287 (11)	0.22 (0.02)	−24 (6)	69 (2)	35 (3)	76 (2)	55 (1)
10 mg/mL	2:1	262 (15)	0.19 (0.03)	−29 (5)	71 (3)	19 (3)	77 (6)	54 (4)
20 mg/mL	2:1	254 (3)	0.22 (0.02)	−27 (7)	69 (5)	25 (6)	69 (2)	50 (1)
30 mg/mL	2:1	248 (25)	0.28 (0.08)	−28 (5)	71 (9)	10 (8)	77 (12)	54 (10)

**Table 4:** Physico-chemical characterization of H-NMeds obtained with the microfluidic device varying the ratio between PLGA and Cholesterol. Standard Deviation (SD) is reported in parentheses.

[Materials]	FRR	PLGA:Chol Ratio	Size (SD)	PDI (SD)	Zeta (SD)	Weight Yield % (SD)	Pluronic % (SD)	Chol Recovery % (SD)	Chol Content % (SD)
20 mg/mL	2:1	0:100	402 (21)	0.24 (0.10)	−24 (4)	64 (6)	20 (10)	37 (3)	67 (5)
20 mg/mL	2:1	25:75	391 (27)	0.20 (0.04)	−28 (6)	54 (4)	21 (5)	35 (4)	48 (6)
20 mg/mL	2:1	50:50	254 (3)	0.22 (0.02)	−27 (7)	69 (5)	25 (6)	69 (2)	50 (1)
20 mg/mL	2:1	75:25	223 (2)	0.26 (0.03)	−27 (6)	70 (4)	22 (10)	82 (6)	29 (2)
20 mg/mL	2:1	100:0	154 (1)	0.22 (0.00)	−32 (5)	82 (4)	20 (9)	/	/

## Data Availability

The data presented in this study are available on request from the corresponding author.
